# *Lhx2* Is Required for Patterning and Expansion of a Distinct Progenitor Cell Population Committed to Eye Development

**DOI:** 10.1371/journal.pone.0023387

**Published:** 2011-08-19

**Authors:** Anna-Carin Hägglund, Lina Dahl, Leif Carlsson

**Affiliations:** Umeå Center for Molecular Medicine, Umeå University, Umeå, Sweden; University of Washington, United States of America

## Abstract

Progenitor cells committed to eye development become specified in the prospective forebrain and develop subsequently into the optic vesicle and the optic cup. The optic vesicle induces formation of the lens placode in surface ectoderm from which the lens develops. Numerous transcription factors are involved in this process, including the eye-field transcription factors. However, many of these transcription factors also regulate the patterning of the anterior neural plate and their specific role in eye development is difficult to discern since eye-committed progenitor cells are poorly defined. By using a specific part of the *Lhx2* promoter to regulate *Cre* recombinase expression in transgenic mice we have been able to define a distinct progenitor cell population in the forebrain solely committed to eye development. Conditional inactivation of *Lhx2* in these progenitor cells causes an arrest in eye development at the stage when the optic vesicle induces lens placode formation in the surface ectoderm. The eye-committed progenitor cell population is present in the *Lhx2^−/−^* embryonic forebrain suggesting that commitment to eye development is Lhx2-independent. However, re-expression of Lhx2 in *Lhx2^−/−^* progenitor cells only promotes development of retinal pigment epithelium cells, indicating that Lhx2 promotes the acquisition of the oligopotent fate of these progenitor cells. This approach also allowed us to identify genes that distinguish *Lhx2* function in eye development from that in the forebrain. Thus, we have defined a distinct progenitor cell population in the forebrain committed to eye development and identified genes linked to Lhx2's function in the expansion and patterning of these progenitor cells.

## Introduction

The vertebrate eye is a complex and highly specialised neurosensory organ that converts light (photons) into electro-chemical pulses that the brain can translate into images. The development of the eye proceeds through co-ordinated interactions between tissues of different embryonic origin. Immediately after initiation of gastrulation, the eye field is specified in the anterior neural plate [1]. The first morphological sign of eye differentiation is the formation of the optic sulci, which are bilateral indentations in the eye field [2]. This is followed by an evagination of the lateral walls of the diencephalon, giving rise to the optic vesicle [2]. Subsequent interactions between the optic vesicle and the surface ectoderm initiate the formation of the lens placode in the ectoderm and mutual interactions between the lens placode and the optic vesicle promote the formation of the optic cup [3]. Lens morphogenesis, establishment of dorso-ventral polarity of the optic vesicle, and pattering of the optic cup into the retina, retinal pigmented epithelium (RPE) cells and optic stalk occur concurrently with these events [3].

The development of the eye is regulated by a number of signalling pathways active at different time points during morphogenesis [4]. Initially, the eye field is induced by the non-canonical Wnt pathway [5], whereas inhibition of the canonical Wnt/β-catenin is required for the differentiation of the eye field from the diencephalic region [1]. The segregation of the eye field into two domains has been proposed to be mediated by the ventralising properties of the hedgehog signalling pathway [6], [7]. Following the transformation of the optic vesicle into the optic cup, the opposing actions of bone morphogenetic protein (Bmp) and hedgehog signalling are thought to generate dorso-ventral patterning. Hedgehog signalling has been implicated in the specification of ventral structures such as the optic stalk, and the retinotectal projection of the retinal axons [8], whereas Bmp signalling has also been shown to be involved in optic vesicle development and lens placode induction [9], [10], [11], [12]. The specification of the early neural retina is mediated by fibroblast growth factor (Fgf) emanating from the surface ectoderm in the prospective lens placode [13], whereas the RPE cells have been suggested to be specified by the transforming growth factor β (Tgfβ) family member Activin A which is secreted by the extraocular mesenchyme [14].

These patterning morphogens impose their actions by activating a cascade of transcription factors that establish cellular identity and subsequent interactions with the environment. The expression of a combination of transcription factors in cells in the anterior neural plate defines the eye field. These transcription factors are collectively referred to as the eye field transcription factors and include *Lhx2*, *Pax6*, *Rx*, *Tlx*, *Six6*, *Six3*, and *ET* [15], and most of these transcription factors play an important role in eye development [16], [17], [18], [19], [20], [21]. Different domains in the developing eye express distinct transcription factors as each structure of the eye become specified. Most of these transcription factors are also important for the specification of the structure they are expressed in. In the early optic cup stage, most cells initiate the expression of *Mitf* whereas cells in close proximity to the lens placode, receiving Fgf signalling, start to express *Vsx2* (also *Chx10*). *Vsx2* represses *Mitf* expression which specifies the neural retina [13], whereas the cells located further away from the lens placode maintain *Mitf* expression specifying the RPE cells [13], [14]. The cells in the optic stalk in the ventral part of the optic cup express *Pax2* [22], [23], and formation of the lens placode in the surface ectoderm is characterised by the expression of *Sox2*, *Pax6* and *Six3* [11], [24], [25].

Importantly, many of the eye field transcription factors have additional function(s) in the forebrain neuroectoderm as revealed by defective forebrain development in the respective homozygous mutant mouse embryos [17], [18], [21]. The LIM-homeobox gene *Lhx2* has been shown to be important in the development of the eyes since eye development is arrested at the optic vesicle stage prior to lens placode induction in *Lhx2^−/−^* mouse embryos [18], [26]. A central function of *Lhx2* in eye development has also been revealed by the observation that over-expression of various combination of eye field transcription factors other than *Lhx2* only induce ectopic eyes when endogenous Lhx2 expression is induced [15]. However, *Lhx2* is expressed in the anterior neural plate prior to the formation of the optic vesicle and the *Lhx2* null embryos have severe defects in other forebrain structures, such as the cerebral cortex and the hippocampus, revealing that *Lhx2* is also involved in the patterning of the forebrain [18], [27], [28], [29]. Thus, to fully understand the mechanisms governing eye development it is important to distinguish the function of genes in the initial patterning of the forebrain from that in the expansion and differentiation of eye committed neural ectoderm.

Identification of the cells in the early forebrain solely committed to eye development would be an important step in examining the function of a gene prior to and after commitment to eye development. To elucidate the molecular basis for *Lhx2* function(s) at various stages of eye development it is necessary to distinguish its function prior to and after this commitment step. We therefore developed a novel Cre transgenic mouse strain, denoted *Lhx2-Cre*, where *Cre* expression was regulated by an 11 kb genomic region of the *Lhx2* promoter immediately upstream of the transcriptional start. *Cre* expression was not detected in all *Lhx2* expressing cells; it rather defined a progenitor cells in the optic pit of the prospective forebrain at embryonic day 8.25 (E8.25) committed to generate the neural part of the eye. Thus, the *Lhx2-Cre* mouse strain will be a very useful tool to examine the function of genes prior to and after commitment of cells during eye development and in the anterior neural plate. Conditional inactivation of *Lhx2* in the Cre^+^ cells led to a developmental arrest just prior to formation of the optic cup and a subsequent deterioration of the optic vesicle. The optic vesicle developed further in these embryos compared with the conventional *Lhx2^−/−^* embryos. Moreover, genes important for lens differentiation were induced in the surface ectoderm leading to the formation of a lens placode. However, further development of the lens placode required maintained *Lhx2* expression since the lens placode regressed in mutant embryos. *Cre* expression was detected in the optic vesicle in *Lhx2^−/−^* embryos suggesting that commitment to eye development in the neural plate is independent of *Lhx2* expression. Expression of transgenic *Lhx2* in the Cre^+^ cell in the *Lhx2^−/−^* background could partly rescue eye development as only cells of the RPE layer developed in these animals. Furthermore, by analysing the expression pattern of a number of genes putatively regulated by *Lhx2* in a different cellular context [30], we identify genes that may potentially be linked to *Lhx2* function also in eye development.

## Results

### Cre expression in the *Lhx2-Cre* transgenic mouse strain defines a distinct progenitor cell population in the anterior neural plate committed to the development of the neural part of the eye

During early development the *Lhx2* gene is widely expressed in the prospective forebrain including the optic vesicle/cup, neural retina, optic stalk and RPE cells [28], [31] (Figure S1A), suggesting that regulatory regions of the Lhx2 gene direct expression to the forebrain as well as to the developing eye. To determine if it is possible to define regulatory regions directing expression to the developing eye we used various parts of the *Lhx2* promoter to drive expression *Cre* recombinase in transgenic mice. We initially used a 5 kb and an 11 kb genomic region immediately upstream of the Lhx2 transcriptional start site to drive expression of the *Cre*. We crossed the *Lhx2-Cre* transgenic mice to the *ROSA26 Reporter* (*ROSA26R*) mouse strain to generate *Lhx2-Cre:ROSA26R* double transgenic mice. These mice enabled the detection of expression and functional activity of Cre recombinase and therefore permitted lineage tracing of *Cre* expressing cells based on β-Galactosidase (β-Gal) activity [32]. No reproducible expression pattern could be detected when the 5 kb region was used to drive expression of *Cre* in transgenic mice (data not shown). However, when the 11 kb region was used to drive *Cre* expression in the double transgenic animals we reproducible observed β-Gal^+^ cells in the neural part of the eye (i.e. retina, RPE cells and optic stalk), whereas very few β-Gal^+^ cells could be detected in the developing forebrain where the endogenous *Lhx2* gene is highly expressed (Figure S1B). The Lhx2-Cre transgene was also expressed in other cells of neural origin where the endogenous Lhx2 gene is expressed (Figure S1D,E,F,G). Since an additional independently generated *Lhx2-Cre* transgenic founder mouse strain showed a similar distribution of β-Gal^+^ cells in the eye suggested that the 11 kb fragment contained a regulatory region that directed gene expression to the neural part of the developing eye that was relatively unaffected by positional effects putatively imposed by the genomic insertion site (Figure S1C). One of these *Lhx2(11kb)-Cre* transgenic founder mouse strains (Figure S1B), referred to as the *Lhx2-Cre* mouse strain, was selected for further experiments.

**Figure 1:**
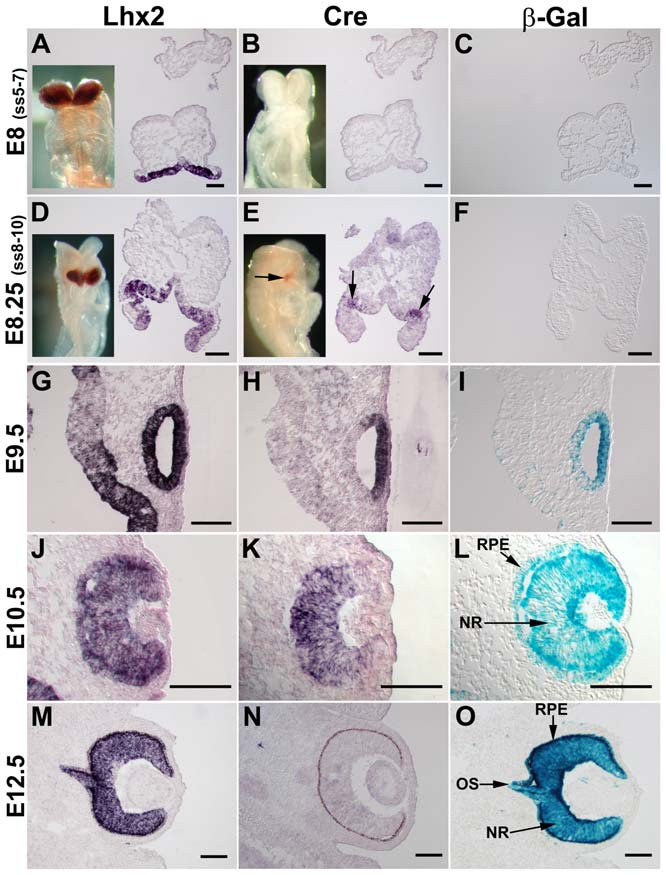
Cre expression in the *Lhx2-Cre* transgenic mouse strain defines the first progenitor cells committed to eye development in the anterior neural plate. (A–C) In situ hybridisation analyses and β-Gal staining of transversal sections of E8 embryos at somite stage 5–7 (ss5–7) to analyse *Lhx2* expression (A), *Cre* expression (B), and β-Gal activity (C), in the anterior neural plate. In situ hybridisation analysis on whole mount embryos at the same developmental stage are inserted in A and B. (D–F) In situ hybridisation analyses and β-Gal staining of transversal sections of E8.25 (ss8–10) to determine *Lhx2* expression (D), *Cre* expression (E), and β-Gal activity (F), in the anterior neural plate. In situ hybridisation analysis on whole mount embryos at the same developmental stage are inserted in D and E. The distinct Cre^+^ cell population in the lateral part of the optic vesicle are indicated by arrows (E). (G–I) In situ hybridisation analyses and β-Gal staining of transversal sections of E9.5 embryos to analyse *Lhx2* expression (G), *Cre* expression (H), and β-Gal activity (I), in the optic vesicle. (J–L) In situ hybridisation analyses and β-Gal staining of transversal sections of E10.5 embryos to analyse *Lhx2* expression (J), *Cre* expression (K), and β-Gal activity (L), in the optic cup. (M–O) In situ hybridisation analyses and β-Gal staining of coronal sections of E12.5 embryos to analyse *Lhx2* expression (M), *Cre* expression (N), and β-Gal activity (O), in the developing eye. NR, neural retina. RPE, retinal pigment epithelium. OS, optic stalk. Scale bars: 100 µm.

To examine the specificity and the timing of *Cre* expression compared with expression of the endogenous *Lhx2* gene, we analysed *Lhx2* and *Cre* expression, and β-Gal activity during embryonic development of *Lhx2-Cre:ROSA26R* double transgenic animals. The endogenous *Lhx2* gene was expressed in the entire prospective forebrain and in all neural cell types of the eye during embryonic development from E8 (ss5–10) until E12.5 (Figure 1A,D,G,J,M). However, *Cre* expression was first detected in a few cells in the most lateral part of the optic pit at E8.25 (Figure 1E), but was not detected prior to this stage (Figure 1B). *Cre* expression was detected in the prospective neural retina at E9.5 and E10.5 (Figure 1H,K), whereas no *Cre* expression was detected in any part of the eye after E12.5 (Figure 1N). To trace the cells that had expressed Cre we performed staining for β-Gal activity in the *Lhx2-Cre:ROSA26R* double transgenic embryos. We were unable to detect β-Gal^+^ cells prior to E9 including the earliest time point when *Cre* expression was detected (Figure 1C,F), suggesting a slight delay between *Cre* expression and recombination of the *ROSA26* locus. However, β-Gal^+^ cells were reproducibly detected in the optic vesicle at E9.5 (Figure 1I), and all cells of the neural retina, the RPE cells and the optic stalk are β-Gal^+^ at E10.5 and E12.5 (Figure 1L,O). Thus, the cells expressing the *Lhx2-Cre* transgene at E8.25 in the optic pit define progenitor cells in the forebrain solely committed to become the neural part of the eye (Figure 1E).

### Conditional inactivation of *Lhx2* in the eye-committed progenitor cells leads to an immediate arrest in eye development

**Figure 2:**
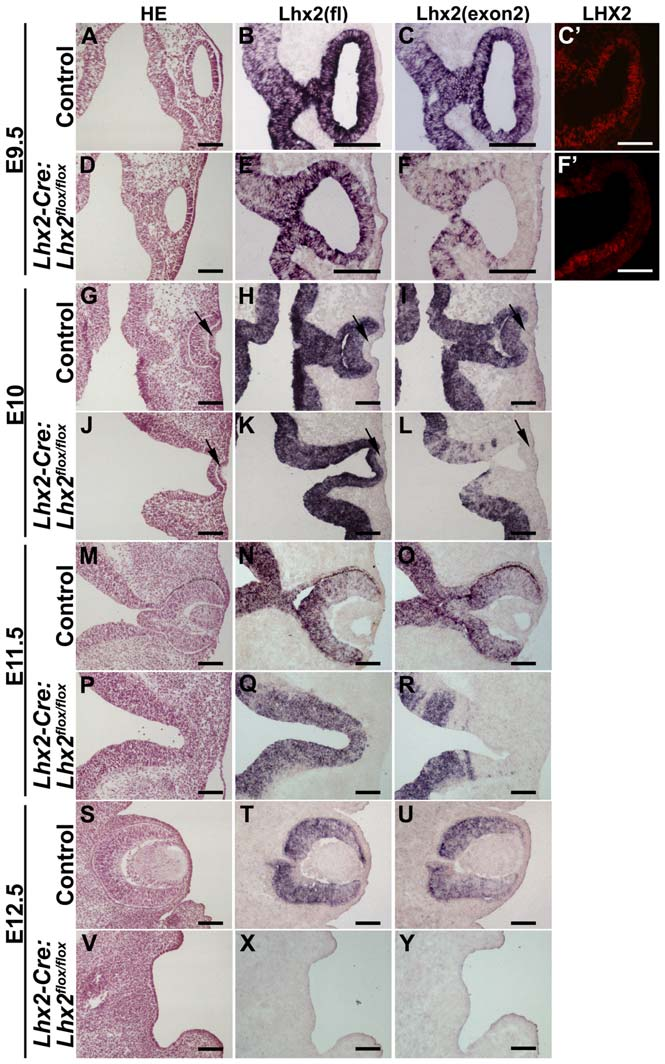
Conditional inactivation of Lhx2 in the eye-committed progenitor cells leads to an immediate arrest in eye development. (A–F) Analyses of transversal sections of optic vesicles in E9.5 control (A–C) and mutant (*Lhx2-Cre:Lhx2^flox/flox^*) (D–F) embryos. Hematoxylin/Eosin (HE) staining (A,D), in situ hybridisation analyses using a full-length (fl) *Lhx2* probe (B,E), and a probe specific for exon 2 (exon2) (C,F). Wild-type *Lhx2* mRNA hybridizes to both the fl and the exon 2 probe whereas mutant mRNA only hybridizes to the fl probe. (C′,F′) Immunohistochemical analysis on coronal sections of control (C′) and mutant optic vesicle (F′) confirming that no Lhx2 protein is detected in cells expressing mutant mRNA. Transversal (G–R) and coronal (S–Y) sections of embryos at the indicated developmental stages comparing eye development and expression of wild-type and mutant *Lhx2* mRNA in control embryos to embryos where *Lhx2* has been inactivated in the eye committed progenitor cells. Arrows indicate lens placode formation in the control and mutant embryos. Scale bars: 100 µm.

This transgenic mouse strain will be a useful tool for elucidating the function of genes during eye development without affecting the patterning of the forebrain. The *Lhx2* gene is expressed in the entire forebrain region prior to formation of the optic pit and is subsequently expressed in the neural part of the eye during the differentiation process [33] (Figure 1A,D,G,J,M). Eye development in *Lhx2^−/−^* embryos is arrested at the optic vesicle stage [18]. However, it is not clear whether this phenotype is caused by defects in the commitment, or expansion/patterning step, or both. Since it is not possible to address these issues in the conventional mutant, we used the *Lhx2-Cre* mouse strain to conditionally inactivate *Lhx2* selectively in the eye committed progenitor cells by crossing the *Lhx2-Cre* mouse strain to a mouse strain with “floxed” *Lhx2* alleles (*Lhx2^flox/flox^*) [34]. All adult *Lhx2-Cre:Lhx2^flox/flox^* mice obtained were anophthalmic and histological sections of newborn mice revealed that all eye structures were lacking in the *Lhx2-Cre:Lhx2^flox/flox^* mice (Figure S2), supporting a role for *Lhx2* in eye development in patterning and/or expansion of eye-committed progenitor cells. We next wanted to analyse the efficiency of *Lhx2* inactivation and at what stage eye development was arrested in the conditional mutant embryos. Exon 2 of the *Lhx2* gene is deleted in the conditional mutant and we can therefore distinguish cells expressing the mutant allele from those expressing the normal allele since mRNA expressed from the latter hybridise to both a full length probe as well as a probe containing only exon 2, whereas mRNA expressed from mutant allele only hybridise to the full length probe [34]. *Cre* is expressed in the eye committed progenitor cells at E8.5 and at E9 (ss10–15) we reproducibly noticed a few cells with an inactivated *Lhx2* gene in the optic vesicle (data not shown), and at E9.5 (ss24–28) the *Lhx2* gene has been efficiently inactivated in the entire optic vesicle (Figure 2B,C,E,F). Deletion of exon 2 leads to an almost immediate down-regulation of Lhx2 protein in these cells (Figure 2 C′,F′). At this stage the control and mutant optic vesicles are still morphologically indistinguishable (Figure 2A,D), although an increased number of apoptotic cells could be observed in the conditional mutant already at this stage (Figure S3). However, at E10 the optic vesicle has differentiated into the optic cup in control embryos whereas this process is blocked in the conditional mutant (Figure 2G,J). While the *Lhx2* mRNA is expressed in both the control and mutant optic vesicle it is efficiently and reproducibly inactivated in the entire optic vesicle in the *Lhx2-Cre:Lhx2^flox/flox^* animals (Figure 2H,I,K,L). Eye development proceeds slightly longer in the conditional mutant compared with the conventional (*Lhx2^−/−^*) mutant since the optic vesicle in the conditional mutant comes in close contact with the surface ectoderm and appears to induce a thickening of the surface ectoderm indicative of lens placode formation (Figure 2J,K,L) [18], [26]. Inactivation of the *Lhx2* gene in eye committed progenitor cells led to a degeneration of the optic vesicle at E11.5 (Figure 2M,P), and at E12.5 all neural structures of the eye were absent and no discernible lens structure could be identified (Figure 2S,V). Similar to the control eye, *Lhx2* mRNA is expressed in the arrested optic vesicle and cells expressing the mutated allele are maintained in its most lateral part at E11.5 and no cells expressing the control mRNA are present in this area (Figure 2N,O,Q,R). Thus, although Cre is transiently expressed, the complete block in eye development suggests that all eye-committed progenitor cells are developmentally arrested in the mutant and appear to die by apoptosis. All neural parts of the eye normally express *Lhx2* at E12.5 but no cells expressing *Lhx2* mRNA were evident in this region at this stage, confirming the complete degeneration of all eye-associated structures derived from the optic vesicle (Figure 2T,U,X,Y).

### Early pattering of the optic vesicle and the lens placode is initiated prior to their developmental arrest and degeneration following conditional inactivation of *Lhx2*

**Figure 3:**
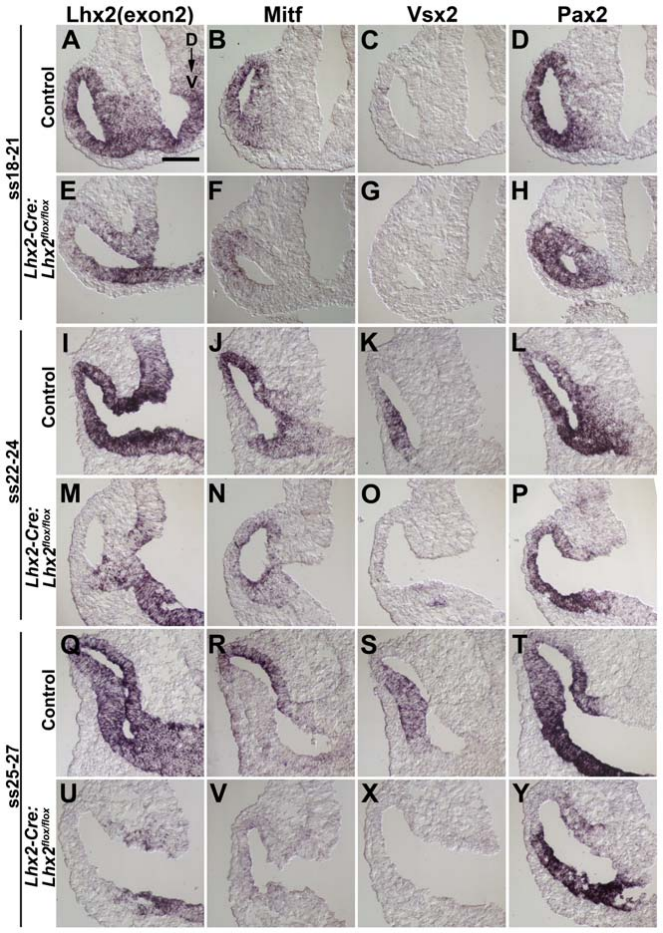
Early patterning of the optic vesicle is initiated following conditional inactivation of *Lhx2* in the eye-committed progenitor cells. (A–Y) In situ hybridisation analyses of coronal sections of the optic vesicles in control and mutant (*Lhx2-Cre:Lhx2^flox/flox^*) embryos at the indicated developmental stages. A,E,I,M,Q,U are in situ hybridisation analyses to detect exon 2 (exon2) in the *Lhx2* mRNA to reveal the domain in the optic vesicle where the *Lhx2* gene has been inactivated. B,F,J,N,R,V are in situ hybridisation analyses to detect expression of the RPE cell-specific gene *Mitf*, which is expressed in the entire optic vesicle in early development and eventually becomes RPE cell-specific. C,G,K,O,S,X are in situ hybridisation analyses to detect expression of neural retina specific gene *Vsx2*. D,H,L,P,T,Y are in situ hybridisation analyses to detect expression of the optic stalk-specific gene *Pax2*. Dorsal to Ventral orientation for all sections is indicated in A. Scale bar: 100 µm.

To determine how the early patterning of the optic vesicle into prospective RPE cells, neural retina and optic stalk was affected by the conditional inactivation of *Lhx2*, we analysed the expression of *Mitf*, *Vsx2*, and *Pax2*, respectively. *Lhx2* is efficiently and consistently inactivated from an early time point beginning at ss18–22 and onwards (Figure 3A,E,I,M,Q,U). Expression of *Mitf* is initially detected in the mutant optic vesicle similar to the control but is subsequently down-regulated in the mutant optic vesicle (Figure 3B,F,J,N,R,V). Expression of the neural retina-specific gene *Vsx2* is not detected at any stage in the mutant optic vesicle (Figure 3C,G,K,O,S,X). *Pax2* is expressed in the mutant optic vesicle and appears to become regionalised to the prospective optic stalk in a similar manner as the control optic vesicle (Figure 3D,H,L,P,T,Y). These results support the notion that eye development proceeds further in the conditional mutant compared with the conventional mutant, since neither *Mitf* nor *Pax2* are expressed in the optic vesicle of the conventional mutant [26]. These data suggest that the patterning of the conditional mutant optic vesicle is initiated prior to its developmental arrest and subsequent degeneration.

**Figure 4:**
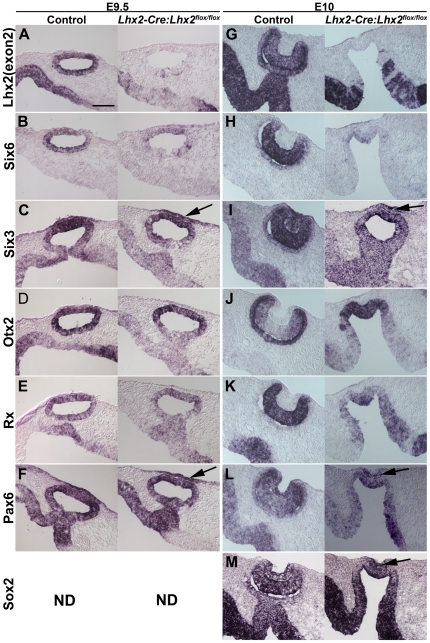
The optic vesicle and the lens placode develop further in the conditional mutant compared with conventional mutant. Gene expression is analysed in optic vesicles in transversal sections at E9.5 (A–F) in control embryos (left panels) and in the conditional mutants (*Lhx2-Cre:Lhx2^flox/flox^*) (right panels). Gene expression is analysed in optic cups on transversal sections at E10 (G–M) in control embryos (left panels) and in the conditional mutants (right panels). In situ hybridisation analyses to detect exon 2 (exon2) of the *Lhx2* gene to reveal the domain in the optic vesicle where the *Lhx2* gene has been inactivated in the conditional mutant at E9.5 (A) and at E10 (G). In situ hybridisation to analyse expression of: *Six6* in the control and conditional mutant at E9.5 (B) and at E10 (H), *Six3* in the control and conditional mutant at E9.5 (C) and at E10 (I), *Otx2* in the control and conditional mutant at E9.5 (D) and at E10 (J), *Rx* in the control and conditional mutant at E9.5 (E) and at E10 (K), *Pax6* in the control and conditional mutant at E9.5 (F) and at E10 (L), and *Sox2* in the control and conditional mutant at E10 (M). *Six6* expression is down-regulated both at E9.5 (B) and E10 (H). The thickening of the surface ectoderm acquires some molecular characteristics of the lens placode as determined by expression of *Six3* (arrows, C and I), *Pax6* (arrows, F and L) and *Sox2* (arrows, M). ND, not done. Scale bar: 100 µm.

To examine potential immediate consequences of *Lhx2* inactivation in eye committed progenitor cells, we analysed the expression of various transcription factors, including the eye field transcription factors, involved in the early steps of optic vesicle and lens formation. At E9.5 when the *Lhx2* gene is completely inactivated in the optic vesicle but the gross morphology of the control and the mutant optic vesicle is indistinguishable (Figure 4A and Figure 2A,D), expression of *Six6* is already significantly down regulated in mutant optic vesicle (Figure 4B), whereas expression of *Six3*, *Otx2*, *Rx* and *Pax6* were not significantly different between control and mutant optic vesicles (Figure 4C–F). This observation is in agreement with the notion that *Lhx2* cooperates with *Pax6* to induce *Six6* expression [33]. At this stage the cells in the adjacent surface ectoderm express *Six3* and *Pax6* in both control and mutant optic vesicles (Figure 4C,F), suggesting that the thickening of surface ectoderm induced in the conditional *Lhx2* mutant also acquire lens placode fate at the molecular level (Figure 2J–L). It has been suggested that the optic vesicle can initiate lens placode identity at E9.5 in the conventional *Lhx2^−/−^* mutant but not maintain it as *Six3* and *Sox2* expression is lost at E10 [26]. We therefore analysed gene expression at E10 in these mutants. *Lhx2* is efficiently and reproducibly inactivated in the lateral part of the optic vesicle at this stage (Figure 4G) and *Six6* expression was significantly attenuated at this stage (Figure 4H). However, although slightly reduced, *Six3*, *Otx2*, *Rx*, *Pax6* and *Sox2* were still expressed in the developmentally arrested mutant optic vesicle at this stage (Figure 4I–M). Furthermore, the mutant optic vesicle induced and maintained the expression of many lens-specific transcription factors in the surface ectoderm such as *Six3*, *Pax6* and *Sox2* (Figure 4I,L,M), further supporting the observation that a lens placode is formed in these mutants. However, when the optic vesicle deteriorates the putative lens placode does not develop further although it has acquired both morphological and molecular characteristics of a lens placode.

### Differential regulation of *Bmp4* and *Bmp7* in the eye-committed progenitor cells

**Figure 5:**
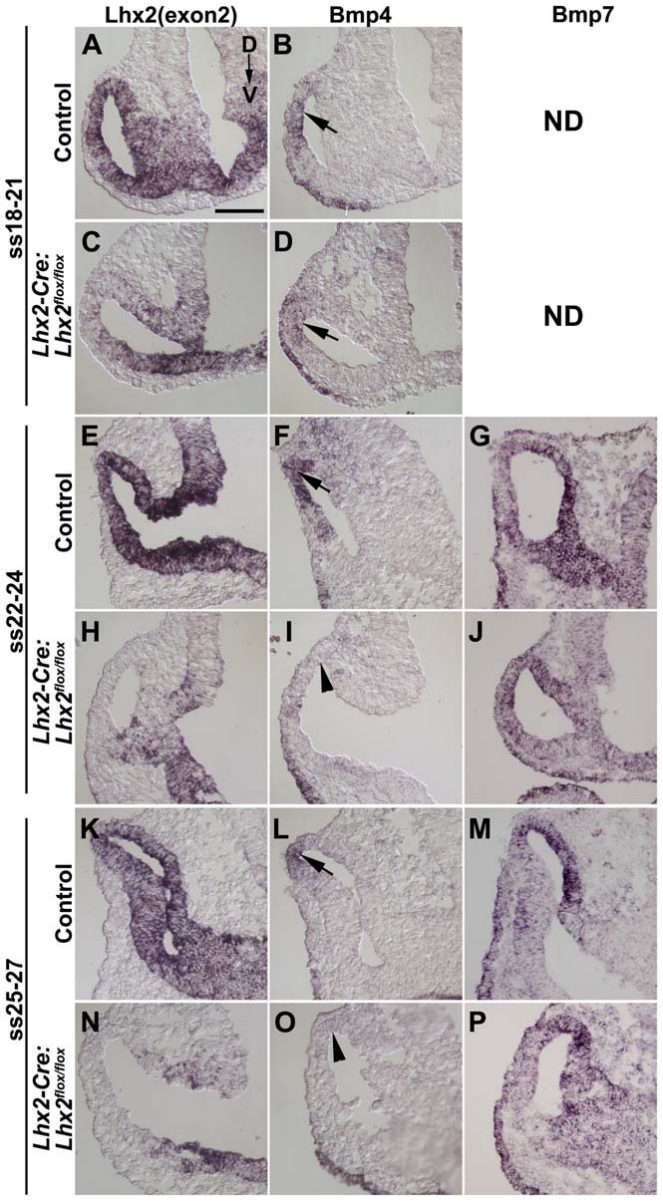
Differential regulation of *Bmp4* and *Bmp7* expression by Lhx2. (A–P) In situ hybridisation analyses of coronal sections of the optic vesicles in control and mutant (*Lhx2-Cre:Lhx2^flox/flox^*) embryos at the indicated developmental stages. A,C,E,H,K,N are in situ hybridisation analyses to detect exon 2 (exon2) in the *Lhx2* mRNA to reveal the domain in the optic vesicle where the *Lhx2* gene has been inactivated. B,D,F,I,L,O are in situ hybridisation analyses to detect expression of *Bmp4*. G,J,M,P are in situ hybridisation analyses to detect expression of *Bmp7*. *Bmp4* expression is initiated dorsally in control embryos (B) and in the conditional mutants (D) at ss18–21 (arrows). *Bmp4* expression is maintained in the controls at later developmental stages (F,L) whereas the expression is down-regulated in the conditional mutants at later developmental stages (I,O, arrow heads). *Bmp7* expression is initiated and maintained in both the control embryos (G,M) and conditional mutants (J,P) at ss22–24 and at ss25–27. ND, not done. Dorsal to Ventral orientation for all sections is indicated in A. Scale bar: 100 µm.

*Lhx2* function has been suggested to be required for Bmp signalling since *Bmp4* and *Bmp7* are not expressed in the *Lhx2^−/−^* optic vesicle [26]. Since *Bmp7* and *Bmp4* have been shown to be important for optic vesicle and lens placode development, respectively [9], [11], we analysed *Bmp4* and *Bmp7* expression in the optic vesicles immediately after *Lhx2* had been conditionally inactivated in the early eye committed progenitor cells. Conditional inactivation of *Lhx2* was efficient in the distal part of the optic vesicle at this stage (Figure 5A,C,E,H,K,N). *Bmp7* expression was readily detected in both the control and mutant optic vesicles from E9 (ss22–24 and ss25–27 embryos) (Figure 5G,J,M,P). *Bmp4* expression was initiated in the dorsal part of the mutant optic vesicle similar to the control optic vesicle at early E9 (ss18–21) (Figure 5B,D). However, by ss22–24 *Bmp4* expression is down-regulated in the mutant optic vesicle whereas its expression was maintained in the dorsal part of the control optic vesicle (Figure 5F,I,L,O). These results suggest that *Bmp4* expression requires maintained *Lhx2* expression in eye committed progenitor cells whereas *Bmp7* expression appears to be independent of maintained *Lhx2* expression in these progenitor cells. The conditional mutant phenotype cannot be solely due to lack of BMP signalling since *Bmp7* expression is maintained in the conditional mutant embryos and addition of BMP7 and BMP4 cannot rescue the *Lhx2^−/−^* phenotype [26]. Thus, additional genes/pathways must contribute to *Lhx2* function in eye development.

### Identification of novel genes putatively linked to Lhx2's function in the optic vesicle

**Figure 6:**
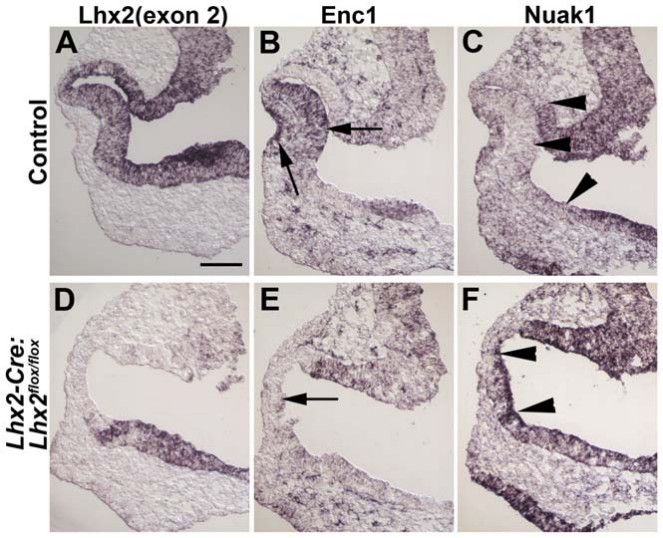
Identification of novel genes putatively linked to *Lhx2* function in eye development. Gene expression analyses are performed at E10 in control animals (A–C) and in conditional mutants (D–F) on coronal sections. In situ hybridisation analyses to detect exon 2 (exon2) of the *Lhx2* gene in the control embryo (A) and in embryos where *Lhx2* has been conditionally inactivated (D) to reveal the domain where the *Lhx2* gene has been inactivated. In situ hybridisation analyses to detect expression of *Enc1* in the control embryo (B) and in the conditional mutant embryo (E). *Enc1* is expressed in the prospective neural retina and lens placode and this expression is not detected in the mutant optic vesicle (arrows). In situ hybridisation analyses to detect *Nuak1* expression in the control embryo (C) and in the conditional mutant embryo (F). *Nuak1* expression is excluded from the optic vesicle in the control embryos whereas its expression extends into this domain in the mutant optic vesicle (arrow heads). Scale bar: 100 µm.

To further elucidate the molecular basis of the *Lhx2* mediated function in eye development, we tried to identify novel genes/pathways putatively linked to *Lhx2* function. To achieve this we took advantage of a previous global gene expression analysis comparing Lhx2^+^ stem cells to their Lhx2^−^ progeny in a different cellular context [30]. Several of the putative *Lhx2* target genes identified in this screen had gene expression pattern in vivo in various organs that overlapped with that of *Lhx2*, suggesting partly overlapping mechanisms for *Lhx2* function in different tissues. Two of these genes, *Enc1* and *Nuak1*, had an expression pattern consistent with this assumption in eye development. Both were expressed in *Lhx2* expressing domains, *Enc1* was expressed in the prospective neural retina and lens whereas *Nuak1* was expressed in the prospective forebrain, but *Nuak1* was excluded from the optic cup (Figure 6A–C). No *Enc1* expression was detected in the mutant optic vesicles in the domain where the *Lhx2* gene has been inactivated and in the lens placode (Figure 6D,E), suggesting that the *Enc1* gene is regulated by *Lhx2* by both cell autonomous and non-autonomous mechanisms. The cell nonautonomous mechanism can be mediated in part by BMP signalling and it has been suggested that *Fgf15* is downstream target of BMP signalling that is mainly mediated by BMP4 [26], [35]. We therefore wanted to determine if the selectively down-regulated expression of *Bmp4* also affected expression of *Fgf15* expression in the conditional mutant optic vesicle. *Fgf15* expression was significantly down-regulated in the mutant optic vesicle (Figure S4), further supporting the idea that *Fgf15* is mainly a downstream target of BMP4 signalling in the neural retina. In contrast, *Nuak1* expression extended into the domain from where it was excluded in the control when *Lhx2* was inactivated (Figure 6F), suggesting that *Lhx2* activate *Nuak1* expression in the prospective forebrain whereas it is suppressing its expression in the optic vesicle/cup. Taken together, the down-regulated expression of *Enc1*, *Six6*, *Fgf15* and *Bmp4* in the optic vesicle, down-regulated expression of *Enc1* in the lens placode and the mis-expression of *Nuak1* in the optic vesicle might contribute to the complete deterioration of both the optic vesicle as well as the lens in the conditional *Lhx2* mutant.

### *Lhx2* expression in the anterior neural plate is not required for commitment to the eye-specific progenitor cell

**Figure 7:**
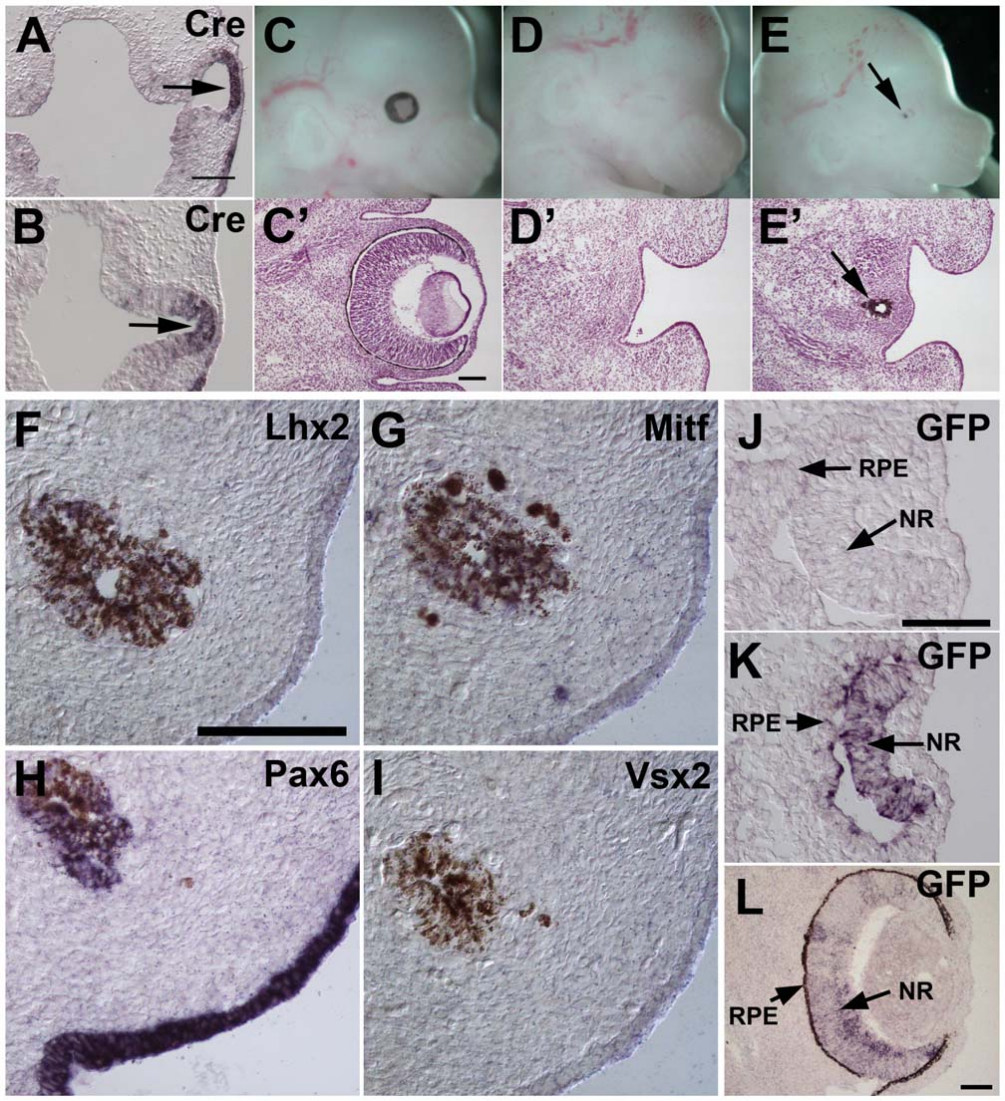
Commitment to the eye-specific progenitor cell is independent of *Lhx2* expression. In situ hybridisation analyses of transversal sections reveal *Cre* expression in the lateral part of the optic vesicle in both control embryos (A) and in Lhx2^−/−^ embryos (B) at E9.5 (arrows). Lateral view of E14.5 embryos (C–E) and HE staining of transversal sections of the head of the same embryos (C′–E′). Eye development is unaffected by transgene expression (*Lhx2-Cre* or *Z/Lhx2-GFP*) in *Lhx2^+/−^* or *Lhx2^+/+^* mice (C,C′, an *Lhx2-Cre:Lhx2^+/−^* embryo). All embryos with an *Lhx2^−/−^* genotype expressing one of the transgenes (*Lhx2-Cre* or *Z/Lhx2-GFP*) are anophthalmic (D,D′, a *Z/Lhx2-GFP:Lhx2^−/−^* embryo). Expression of *Lhx2* in the *Lhx2^−/−^* eye-committed progenitor cells partly rescues eye development since a bilaterally located small mass of pigmented cells appear in all *Lhx-Cre:Z/Lhx2-GFP:Lhx2^−/−^* embryos (E,E′, arrows). In situ hybridisation analyses on transversal sections to detect *Lhx2* expression (F), *Mitf* expression (G), *Pax6* expression (H) and *Vsx2* expression (I) in the pigmented cells that developed in the *Lhx-Cre:Z/Lhx2-GFP:Lhx2^−/−^* embryos. The pigment is brown whereas the in situ hybridisation signal is dark-blue. In situ hybridisation to detect *GFP* expression and hence confirm transgenic *Lhx2* expression in the eye of *Lhx-Cre:Z/Lhx2-GFP* control double transgenic embryos both at E10.5 (K, *Lhx-Cre:Z/Lhx2-GFP:Lhx2^+/+^*) and E14.5 (L, *Lhx-Cre:Z/Lhx2-GFP:Lhx2^+/+^*), whereas *GFP* is not expressed in *Z/Lhx2-GFP* single transgenic embryos (J). NR, neural retina. RPE, retinal pigment epithelium. Scale bars: A–B, C′–E′, F–I, J–L, 100 µm.

Another important aspect of *Lhx2* function is its role in the commitment to eye development as it is widely expressed in the prospective forebrain prior to this step. An advantage with the *Lhx2-Cre* mouse line is that it possible to address what role various genes have in the commitment process since one can use *Cre* expression as a marker for eye commitment, and hence analyse different mutant mice for *Cre* expression in the forebrain. To address this issue we crossed the *Lhx2-Cre* transgenic mouse strain to the conventional *Lhx2* mutant mouse strain to generate *Lhx2-Cre:Lhx2^−/−^* embryos. Analysis of *Cre* expression in such embryos would allow us to assess if *Lhx2* expression is required for commitment to the eye-specific progenitor cell. *Cre* expression was detected in the most lateral part of the optic vesicle at E9.5 in the *Lhx2-Cre:Lhx2^−/−^* mice similar to *Cre* expression in the control embryos (Figure 7A,B), suggesting that commitment to the eye-specific progenitor cell is independent of prior *Lhx2* expression. Moreover, although commitment to eye development had occurred in the *Lhx2^−/−^* forebrain, does not necessarily imply that these progenitor cells also have acquired full competence to generate all parts of the eye derived from the optic vesicle. To address this issue we devised a strategy where it would be possible to rescue eye development by expressing transgenic *Lhx2* in *Lhx2^−/−^* eye-committed progenitor cells. To obtain such mouse strain we used a double reporter transgenic mouse strain (*Z/Lhx2-GFP*), where Cre-mediated recombination induces *Lhx2-GFP* expression (Figure S5). [34]). The *Z/Lhx2-GFP* and the *Lhx2-Cre* transgenes were crossed into the *Lhx2* mutant background. In this cross the *Lhx2-Cre:Z/Lhx2-GFP* double transgenic mice offspring would induce *Lhx2-GFP* expression in Cre^+^ cells. As expected, all *Lhx2^+/−^* or *Lhx2^+/+^* offspring had normal eyes irrespective of their transgenic genotype (Figure 7C,C′), and all single transgenic offspring that were *Lhx2^−/−^* were anophthalmic (Figure 7D,D′). However, in all the *Lhx2-Cre:Z/Lhx2-GFP:Lhx2^−/−^* offspring that we obtained (2) we could observe a bilaterally located small mass of pigmented cells where the eye is normally located (Figure 7E,E′), suggesting a partial rescue of the eye where only the RPE-like cells could form when *Lhx2-GFP* expression was induced in *Lhx2^−/−^* eye progenitor cells. The pigmented cells expressed *Lhx2*, *Mitf* and *Pax6* (Figure 7F–H), whereas expression of the neural retina-specific gene *Vsx2* was not detected (Figure 7I), further supporting the notion that most cells have acquired an RPE cell phenotype. This partial rescue could be due to suboptimal expression of transgenic *Lhx2* expression in the eye committed progenitor cells, or that the *Lhx2* mutant cells are not fully competent for complete eye development upon *Lhx2* re-expression. To address the former point we analysed *GFP* expression in the developing eye in *Lhx2-Cre:Z/Lhx2-GFP* control (e.g. *Lhx2^+/+^* or *Lhx2^+/−^*) mice, which is a reliable indicator for functional Lhx2 expression in *Z/Lhx2-GFP* mice after Cre mediated recombination of the double reporter transgene [34]. Although it is difficult to assess the optimal expression level we could detect wide-spread *GFP* expression in the neural part of the eye at different developmental stages in all *Lhx2-Cre:Z/Lhx2-GFP* embryos analysed (n = 4) (Figure. 7J,K,L), suggesting that transgenic Lhx2 expression was induced in the correct cells at the correct time. Thus, although commitment to eye development occurs in the *Lhx2^−/−^* forebrain, re-expression of *Lhx2* in the *Lhx2^−/−^* eye committed progenitor cells could only promote development of RPE cells.

## Discussion

By using a defined part of the *Lhx2* promoter to regulate the expression of the *Cre* recombinase we have been able to identify cells in the forebrain solely committed to generate the neural part of the eye, suggesting that these Cre^+^ cells are the earliest cells committed to eye development. Commitment to this progenitor cell fate is independent of prior *Lhx2* expression in the anterior neural plate, and we propose that *Lhx2* promote the acquisition of the oligopotent state of this progenitor cell population in addition to its requirement for the subsequent differentiation of the optic vesicle into the optic cup. Eye development progresses further in the conditional mutant compared to the conventional *Lhx2^−/−^* mutant mice since the optic vesicle comes in direct contact with the surface ectoderm and induces a lens placode. However, immediately after the developmental arrest of the optic vesicle in the conditional mutant, both the optic vesicle and the lens placode degenerate leading to a complete lack of these structures. These result reveal that lens development require continuous interactions between the lens placode and the optic vesicle even if the lens placode has acquired many of its molecular characteristics. We have compared the expression pattern of some genes involved in eye development in the optic vesicle and lens placode between the conditional mutant (*Lhx2-Cre-Lhx2^flox/flox^* results presented herein) and the conventional mutant (*Lhx2^−/−^* from refs. [26], [33] in Table 1. The most obvious difference is the induction of lens-specific genes *Sox2*, *Six3* and *Pax6* in the prospective lens placode in the conditional mutant. However, the observation that some genes are differentially expressed in the optic vesicle, such as *Bmp7* and *Pax2*, suggests that *Lhx2* has different function(s) before and after commitment and that the difference is not solely due to the delayed inactivation of the *Lhx2* gene in the conditional mutant. We have also identified novel genes putatively coupled to *Lhx2* function during eye development that previously have been shown to be linked to *Lhx2* function in another stem/progenitor cell system. This approach also allowed us to distinguish the function of *Lhx2* in the forebrain to that in eye development.

**Table 1:** Comparison of gene expression between *Lhx2-Cre:Lhx2^flox/flox^* and *Lhx2^−/−^* embryos in the optic vesicle and lens placode.

Gene	Optic vesicle		Lens placode	
	*Lhx2-Cre: Lhx2^flox/flox^*	*Lhx2^−/−^*	*Lhx2-Cre: Lhx2^flox/flox^*	*Lhx2^−/−^*
*Sox2*	**+** a	**+**	**+**	**-** b
*Six3*	**+**	**+**	**+**	**-**
*Pax6*	**+**	**+**	**+**	**T** c
*Otx2*	**+**	**+**		
*Rx*	**+**	**+**		
*Bmp7*	**+**	**-**		
*Pax2*	**+**	**T**		
*Bmp4*	**T**	**-**		
*Mitf*	**T**	**-**		
*Six6*	**-**	**-**		
*Vsx2*	**-**	**-**		
*Fgf15*	**-**	**-**		

a Expression is detected.

b Expression is not detected.

c Expression is transiently detected.

Analysis of the conventional *Lhx2* mutant has previously shown that *Lhx2* is important for the optic vesicle to optic cup transformation [18], [26]. However, since *Lhx2* is expressed in the entire prospective forebrain prior to optic vesicle formation it is difficult to discern if *Lhx2* has a function in the patterning of the forebrain and hence commitment to eye development, or expansion and patterning of the optic vesicle, or both. This is also pertinent to other transcription factors suggested to have a role in eye development such as *Pax6*, *Six3*, *Six6* and *Tlx* [19], [20], [36], [37]. By using the *Lhx2-Cre* transgenic mouse strain that defines the first progenitor cells committed to generate the neural part of the eye in the anterior neural plate it is possible to molecularly define the role of any gene in patterning/commitment to eye development and subsequent expansion/patterning of the eye committed progenitor cells. We could confirm that *Lhx2* is required in the optic vesicle to optic cup transformation since development is blocked almost immediately following inactivation of *Lhx2* in the optic vesicle. This observation is also in agreement with the finding that induction of eye development by ectopic expression of eye field transcription factors can only occur when endogenous *Lhx2* expression is induced [15]. However, the optic vesicle in the conditional mutant induces formation of a lens placode suggesting that it develops further compared to the optic vesicle in the conventional mutant [18], [26]. Moreover, the patterning process of the optic vesicle is partially and transiently established in the conditional mutant since *Pax2* expression is induced and *Mitf* expression is transiently induced, whereas this process is completely blocked in the *Lhx2^−/−^* embryos. This observation suggests that *Lhx2* is important for both establishing and maintaining the patterning of the optic vesicle. The earliest molecular consequence of inactivation of *Lhx2* in the optic vesicle is the down-regulated expression of *Six6*, whereas expression of most other transcription factors involved in early eye development appears to be unaffected. The down-regulated expression of *Six6* is in agreement with the finding that *Lhx2* synergises with *Pax6* to induce *Six6* expression [33]. However, down-regulated *Six6* expression cannot explain the block in eye development since *Six6^−/−^* mice have a relatively mild eye phenotype affecting only the late stages of eye development [20]. Importantly, our results also indicate that *Lhx2* expression is not required for commitment to the eye-specific progenitor cell since Cre expression is also detected in the distal part of the optic vesicle in *Lhx2^−/−^* embryos. However, expression of transgenic *Lhx2* in the eye committed progenitor cells in the *Lhx2^−/−^* background only promote development of RPE cells, suggesting that *Lhx2* expression prior to eye commitment is important for the progenitor cell to acquire its oligopotency. Thus, *Lhx2* might also regulate the establishment of a fully functional eye progenitor cell.

There are several hypotheses of how optic vesicle-surface ectoderm/lens placode interactions regulate eye development. Many of these hypotheses are based on the study of *Pax6*, which is complicated by the fact that it is expressed in cells both in the optic vesicle and the surface ectoderm/lens placode [38]. Depending on model organism used and experimental design it has either been suggested that the optic vesicle and the lens placode are continuously interacting during eye development, or that lens development becomes independent of the optic vesicle/cup when the lens has reached a certain developmental stage [4], [39]. Some clarifications of *Pax6* function, at least in mice, have been obtained by performing tissue-specific inactivation of *Pax6* in surface ectoderm [24]. These experiments have showed that *Pax6* is required cell autonomously in the surface ectoderm for lens development. Furthermore, the developing lens is not necessary for the formation of the neural retina and RPE layer, but is rather required for the correct organisation and localisation of the neuroepithelium of the eye. Our results reveal that lens placode development is also arrested immediately after *Lhx2* has been inactivated in the optic vesicle. Thus, continuous interactions between the optic vesicle/cup and the developing lens is required for lens formation although the lens placode has formed and acquired many of its molecular characteristics such as expression of *Pax6*, *Six3* and *Sox2* [40].

BMP signalling has been shown to be important for eye development since both *Bmp4* and *Bmp7* mutant mice have profound eye defects [9], [11]. Disrupted BMP signalling has been suggested to contribute to the arrested development of the optic vesicle in the *Lhx2^−/−^* embryos since neither *Bmp4* nor *Bmp7* are expressed and phosphorylated SMADs, the intracellular mediators of BMP signalling, are not detected in mutant optic neuroepithelium [26]. In the conditional mutants expression of *Bmp4* is initiated in the optic vesicle similar to the control animals but is rapidly down-regulated, supporting the notion that *Lhx2* function is partially mediated by BMP signalling. This notion is further supported by the down-regulated expression of Fgf15, which is suggested to be a down-stream target of BMP4 signalling [26], [35]. However, *Bmp7* expression appears to be unaffected in the conditional mutant and hence lack of *Bmp* expression cannot solely explain the *Lhx2* mutant phenotype. Since *Bmp7* has been shown to regulate the expression of *Pax2* explains why *Pax2* expression is unaffected in the conditional mutant whereas it is absent in the *Lhx2^−/−^* mice [26], [41]. The *Bmps* appear therefore to be differently regulated by *Lhx2* where both *Bmp4* and *Bmp7* expression is initiated by *Lhx2* but maintained expression of *Bmp4* is Lhx2-dependent whereas maintained *Bmp7* expression is Lhx2-independent. Moreover, Lhx2 re-expression in the *Lhx2^−/−^* eye committed progenitor cells led to the formation of only RPE cells, which is remarkably similar to the eye phenotype in *Bmp7^−/−^* mice [9]. Since neither *Bmp7* nor *Bmp4* is expressed in the *Lhx2^−/−^* optic vesicle [26], the inability to completely rescue eye development by re-expressing *Lhx2* in the *Lhx2^−/−^* eye progenitor cells might be partly due to suboptimal expression of the *Bmps* when *Lhx2* expression is turned on in the optic vesicle. Alternatively, the level of transgenic *Lhx2* expression might not be sufficient to induce enough *BMP* expression at the correct time in the optic vesicle.

We have previously identified genes putatively linked to *Lhx2* function by comparing global gene expression in Lhx2^+^ progenitor cells to their Lhx2^−^ progeny [30]. Many of these genes showed overlapping gene expression patterns with *Lhx2* in various tissues and progenitor cell populations, suggesting that mediators of *Lhx2* function partly overlap in different tissues/progenitor cell populations. In this study we identified a number of genes that also overlap with *Lhx2* expression during eye development. The gene *Enc1* (Ectodermal neural cortex 1) encodes a Kelch-related protein suggested to be important in the organisation and function of the cytoskeleton [42]. *Enc1* was expressed in the prospective neural retina and the lens placode, and was not detected in these tissues in the conditional *Lhx2* mutant. Since *Lhx2* is expressed in the neural retina but not expressed in the lens placode it suggests that *Lhx2* regulate *Enc1* in neural retina by a cell autonomous mechanism and in the lens placode by a cell nonautonomus mechanism. Putative mediators of the cell nonautonomous regulation of *Enc1* in the lens placode remains to be elucidated, but could partly include mediators of BMP4 signalling since this signalling pathway has been linked to *Lhx2* function in eye development and is important for lens development [11], [26]. A complex regulatory network involving *Lhx2* in eye development is further emphasised by the expression pattern of *Nuak1*, an adenosine monophosphate-activated protein kinase (AMPK)-related kinase (also *Ark5*) suggested to be involved in the regulation of ploidy and senescence [43]. *Nuak1* is normally expressed by the neural ectoderm in the forebrain where *Lhx2* is also expressed, but its expression is excluded in the optic vesicle and its derivatives, e.g. prospective neural retina, RPE cells, and optic stalk. However, in the conditional *Lhx2* mutant the expression domain of *Nuak1* extends into these domains of the developing eye. The most simplistic explanation for this phenotype is that Lhx2 promotes *Nuak1* expression in the forebrain neural ectoderm whereas it suppresses *Nuak1* expression in eye committed neural ectoderm. Thus, the combined effects of down-regulated expression of *Six6*, *Enc1*, *Fgf15* and *Bmp4* in the optic vesicle, misexpression of *Nuak1* in the optic vesicle and down-regulated expression of *Enc1* in the lens placode, might partly explain the developmental arrest and degeneration of the eye in the conditional *Lhx2* mutants. *Lhx2* has been suggested to regulate key determinants of both dorsal and ventral identity [26], and the complex regulation of genes linked to *Lhx2* function presented here starts to explain how *Lhx2* accomplishes that.

Transgenic mice have previously been generated where *Cre* expression is regulated by the promoters of *Crx*, *Rx*, *Pax6* and *Six3* genes directing *Cre* expression to the developing eye [24], [44], [45], [46]. However, the *Rx-Cre* and the *Six3-Cre* transgenic mice also reveal *Cre* expression in neural tissue outside of the eye domain in the forebrain, and the *Pax6-Cre* and *Crx-Cre* mouse strains show restricted expression within the developing eye. To our knowledge the *Lhx2-Cre* mouse strain is the first mouse model where progenitor cells solely committed to generate the neural part of the eye can be identified in the anterior neural ectoderm. Lineage tracing of these cells revealed that they do not contribute to any other cells in the prospective forebrain. The conditional inactivation of *Lhx2* in these eye committed progenitor cells also confirmed this assumption since the forebrain appears to be intact, which is in contrast to the *Lhx2^−/−^* embryos that lack several forebrain structures [18]. Thus, the *Lhx2-Cre* mouse strain will be a very useful tool to elucidate the specific role(s) of any gene in the patterning/commitment of anterior neural plate into eye committed progenitor cells, and the subsequent expansion/pattering of the optic vesicle. Moreover, the role of specific genes in the ability of the optic vesicle to communicate with surface ectoderm and hence induce and promote lens development can also be studied in detail by using the *Lhx2-Cre* mouse strain.

## Materials and Methods

### Ethics statement

The mice were maintained at the animal facility at Umeå University and all experiments involving animals were approved by the local Animal Review Board (approval Ids: A129-10, A31-08 and A31-11).

### Generation and maintenance of mice

The *Lhx2-Cre* transgenic construct was generated by using an 11 kb DNA fragment of the *Lhx2* promoter, which included the first 36 bp of the *Lhx2* coding sequence. The *Lhx2* promoter fragment was fused in-frame with *Cre* recombinase cDNA and a SV40 polyadenylation signal was added. Pronuclear injection of the DNA construct generated two founder lines of which one was chosen for further studies. Generation of *ROSA26R* mice, *Lhx2^−/−^* mice, *Lhx2^flox/flox^* mice and *Z/Lhx2-GFP* transgenic mice has been described previously [18], [32], [34]. The genotype was determined by PCR analysis of genomic DNA extracted from tail biopsies. Primers used to identify the *Lhx2^flox^* allele were: LOX 5′-GCCAGACTAGCAGACGCTGC-3′ and SDL2 5′-CCACCGGTACTCCTCTTCAGAG-3′. Primers used to identify the *Z/Lhx2-GFP* transgene were GFPforward 5′-TTCCACCATATTGCCGTC-3′ and GFPreverse 5′-AGAACTTGCCGCTGTTCA -3′. Primers used to identify the *Lhx2-Cre* transgene were: 1084: 5′-GCGGTCTGGCAGTAAAAACTATC-3′ and 1085: 5′-GTGAAACAGCATTGCTGTCACTT-3′. Primers used to genotype the *ROSA26R* mice were: Lac3 5′-GGT TGT TAC TCG CTC ACA-3′ and Lac4 5′- CGT TAA AGT TGT TCT GCT TC-3′. The morning of the vaginal plug was considered as E0.5.

### Histology, in situ hybridisation and β-Gal staining

Embryos were isolated and fixed in 4% paraformaldehyde (PFA) in PBS at 4°C. Embryos used for β-Gal staining were fixed for 30 minutes and embryos used for in situ hybridisation were fixed for 1–2 hours. After fixation the embryos were transferred to 30% sucrose in PBS for 24 hours at 4°C, mounted in Tissue-tek (Sakura) and stored at −80°C. Sectioning (8–10 µm) was performed on a cryostat (Microm HM505E) and collected on superfrost plus slides (Menzel-Gläser). For hematoxylin-eosin staining, tissue sections were incubated in Mayer's hematoxylin solution for 2 minutes, in water for 15 minutes, in eosin solution for 2 minutes, in 95% ethanol for 2×1 minutes, in 99% ethanol for 2×1 minutes and in xylene for 5 minutes. The slides were mounted with DPX mounting media (VWR). For β-Gal staining, tissue sections were washed for 3×20 minutes in wash buffer (0.1 M phosphate buffer, 2 mM MgCl_2_, 5 mM EGTA, 0.02% NP40 and 0.01% sodium deoxycholate) and subsequently incubated in X-gal buffer (wash buffer supplemented with 1 mg/ml 5-bromo-4-chloro-3-indolyl-D-galactopyranoside (X-gal) (Austral), 5 mM potassium ferrocyanide and 5 mM potassium ferricyanide) over night at room temperature. The reaction was stopped with 3×5 minutes washes with PBS, and sections were mounted in 87% glycerol. Whole-mount in situ hybridisation and in situ hybridisation using DIG labelled probes were performed essentially as previously described [47], [48]. The following probes were used: *Lhx2* (NM_010710, full length cDNA nucleotides 460–1750, exon 2 nucleotides 587–789, *GFP* (hrGFP, complete coding region), *Bmp4* (NM_007554, nucleotides 117–578), *Pax6* (NM_013627, nucleotides 799–1605), *Bmp7* (NM_007557, nucleotides 1–1987), Pax2 (IMAGE clone: 40142573), Cre (Cre recombinase, complete coding region), *Rx* (IMAGE clone: 5366450), *Sox2* (IMAGE clone: 6413283), *Otx2* (NM_144841, nucleotides 338–1158), *Six6* (NM_011384, nucleotides 126–932), *Six3* (BC098096, nucleotides 771–1222), *Vsx2* (IMAGE clone: 6492679), *Mitf* (IMAGE clone: 40047440), *Enc1* (NM_007930, nucleotides 155–2637) and *Nuak1* (NM_001004363, nucleotides 75–2113).

Immunohistochemistry was performed essentially as previously described [49]. Embryos fixed in 4% PFA for 1 hour were sectioned and slides were washed 3×5 minutes in TBS (50 mM Tris-HCl pH 7,4, 150 mM NaCl) and blocked with 10% FCS in TBST (TBS with 0,1% Triton X-100) for 20 minutes. The primary antibodies, rabbit-anti-Lhx2 (dilution 1∶2000) [50] and rabbit-anti-cleaved Caspase-3 (Asp175) (Cell signalling, dilution 1∶1000) diluted in TBST with 5% FCS was applied to slides over night at 4°C. After 3×5 minutes washing in TBST the secondary antibody, Cy3-conjugated donkey anti-rabbit IgG (Jackson ImmunoResearch Laboratories Inc., dilution 1∶1000) was added together with DAPI for 1 hour at room temperature. Slides were subsequently washed 3×5 minutes in TBST before mounting with fluorescence mounting medium (Vectashield, Vector Laboratories).

## Supporting Information

Figure S1 **Lineage tracing of cells reveal that** ***Cre*** **expression is confined to progenitor cells committed to eye development.** In situ hybridisation analyses to detect *Lhx2* expression in the developing eye, forebrain and other cells of neural origin at E12.5 (A), E9.5 (D) and E10.5 (F). β-Gal staining of sections of a head from *Lhx2-Cre:ROSA26R* double transgenic embryo at E12.5 derived from two different *Lhx2-Cre* transgenic founder mouse strains revealing that all neural parts of the eye are β-Gal^+^ in both founder mice (B,C). β-Gal staining of a sagittal section of a whole *Lhx2-Cre:ROSA26R* double transgenic embryo at E9.5 (E) and a transversal section of a head at E10.5 (G). β-Gal^+^ cells can be detected in the midbrain at E9.5 (E, arrow), in the olfactory placode (OE) at E10.5 (G, arrow) and in cells of the hindbrain at E10.5 (G, arrow heads). NR, neural retina. RPE, retinal pigment epithelium. OS, optic stalk. Scale bar: A–E and F–G 500 µm.
(TIF)

Figure S2 **Conditional inactivation of** ***Lhx2*** **in the eye committed progenitor cell population cause anophthalmia.** All adult *Lhx2-Cre:Lhx2^flox/flox^* animals are anophthalmic (C) whereas the *Lhx2^flox/flox^* mice develop normal eyes (A). This phenotype is already manifested at postnatal day 1 since no eye structures can be detected on sections of the head of *Lhx2-Cre:Lhx2^flox/flox^* animals (D) whereas *Lhx2^flox/flox^* animals develop normal eyes (B). Scale bar: 400 µm.
(TIF)

Figure S3 **Increased number of apoptotic cells in the mutant optic vesicle.** Immunohistochemical analysis of coronal sections of control optic vesicle (A) and mutant (*Lhx2-Cre:Lhx2^flox/flox^*) optic vesicle (B) at E9.5 to detect the presence of activated caspase-3. Scale bar: 100 µm.
(TIF)

Figure S4 ***Fgf15*** **expression is significantly down-regulated in the optic vesicle in the conditional mutant.** In situ hybridisation analyses of coronal sections of the optic vesicles in control (A) and mutant (*Lhx2-Cre:Lhx2^flox/flox^*) embryos (B) at E9.5 to detect Fgf15 expression.
(TIF)

Figure S5 ***Lhx2*** **expression is induced following Cre-mediated recombination of the** ***Z/Lhx2-GFP*** **transgene.** Schematic representation of the vector used to generate the *Z/Lhx2-GFP* transgenic mouse strain (upper panel) and the organisation of this vector after Cre-mediated recombination (lower panel). The blue arrows correspond to the mRNA that is generated before and after Cre-mediated recombination of this vector. We utilised an expression system based on the Z/AP double reporter vector developed by Lobe and co-workers [1], where a floxed allele of *β-Geo* (encoding a β-Gal-Neomycin fusion protein) is followed by an expression cassette consisting of the Lhx2 cDNA, an internal ribosomal entry site (IRES) and green fluorescent protein (GFP) cDNA. Thus, cells expressing Cre recombinase will delete the *β-Geo* gene and initiate expression of Lhx2 and GFP since the *Lhx2-GFP* part is placed immediately downstream of the promoter/enhancer. **Supplementary reference.** 1. Lobe, C., Koop, K., Kreppner, W., Lomeli, H., Gertsenstein, M. and Nagy, A. (1999). Z/AP, a double reporter for cre-mediated recombination. Develop. Biol. 208:281–292.
(TIF)
