# Supplementary figures and images for: Lhx2 Is Required for Patterning and Expansion of a Distinct Progenitor Cell Population Committed to Eye Development
Source: PLoS One. 2011 Aug 19;6(8):e23387. doi: 10.1371/journal.pone.0023387 (PMC3158764; doi:10.1371/journal.pone.0023387)

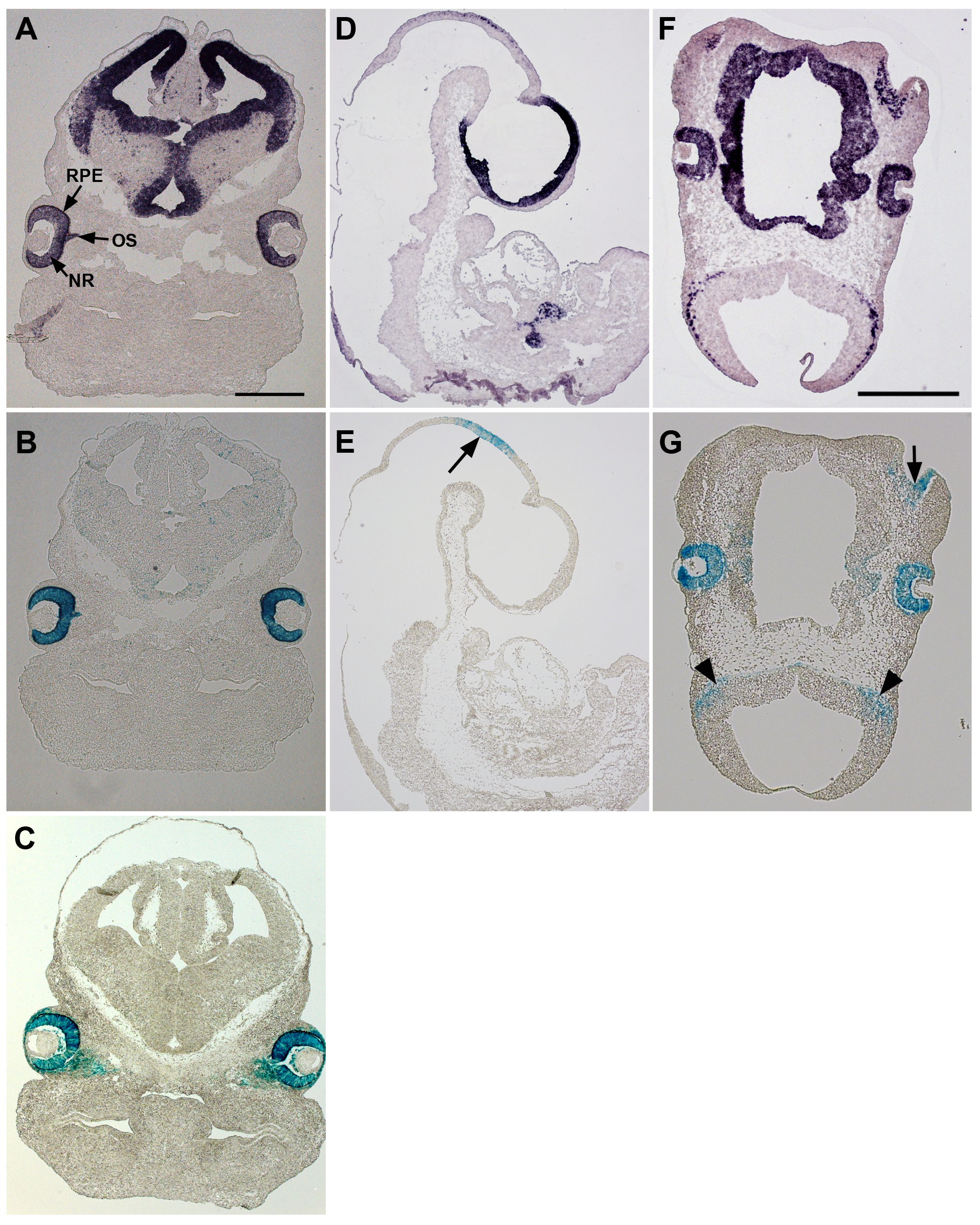

Supplement: Figure S1 — Lineage tracing of cells reveal that Cre expression is confined to progenitor cells committed to eye development. In situ hybridisation analyses to detect Lhx2 expression in the developing eye, forebrain and other cells of neural origin at E12.5 (A), E9.5 (D) and E10.5 (F). β-Gal staining of sections of a head from Lhx2-Cre:ROSA26R double transgenic embryo at E12.5 derived from two different Lhx2-Cre transgenic founder mouse strains revealing that all neural parts of the eye are β-Gal+ in both founder mice (B,C). β-Gal staining of a sagittal section of a whole Lhx2-Cre:ROSA26R double transgenic embryo at E9.5 (E) and a transversal section of a head at E10.5 (G). β-Gal+ cells can be detected in the midbrain at E9.5 (E, arrow), in the olfactory placode (OE) at E10.5 (G, arrow) and in cells of the hindbrain at E10.5 (G, arrow heads). NR, neural retina. RPE, retinal pigment epithelium. OS, optic stalk. Scale bar: A–E and F–G 500 µm. (TIF) [file pone.0023387.s001.tif]

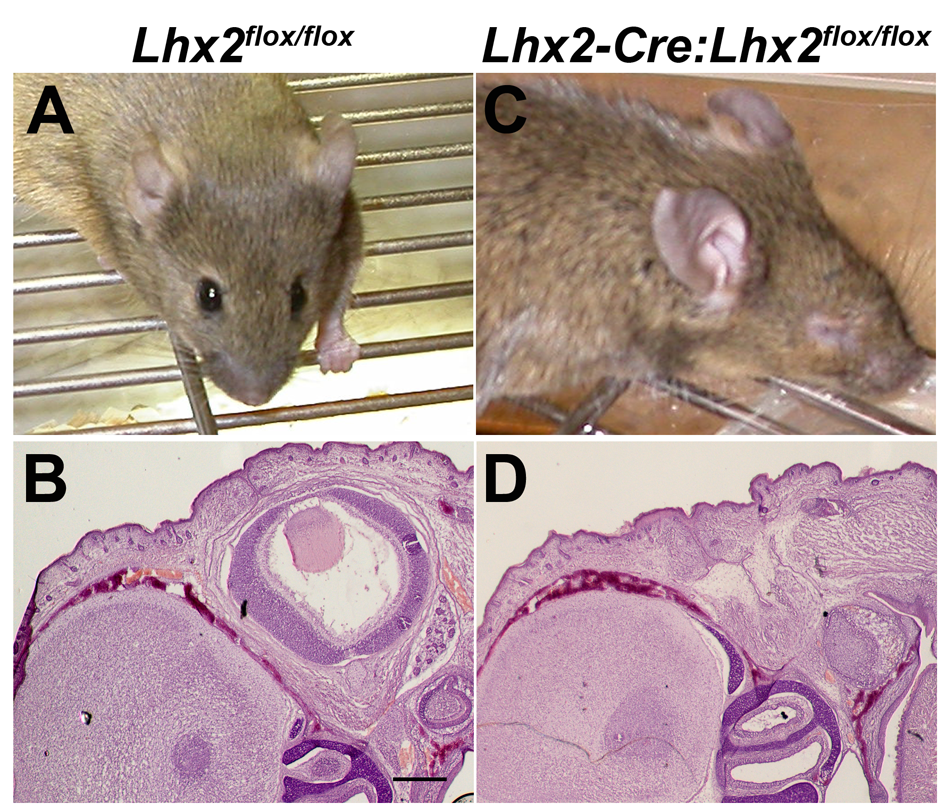

Supplement: Figure S2 — Conditional inactivation of Lhx2 in the eye committed progenitor cell population cause anophthalmia. All adult Lhx2-Cre:Lhx2flox/flox animals are anophthalmic (C) whereas the Lhx2flox/flox mice develop normal eyes (A). This phenotype is already manifested at postnatal day 1 since no eye structures can be detected on sections of the head of Lhx2-Cre:Lhx2flox/flox animals (D) whereas Lhx2flox/flox animals develop normal eyes (B). Scale bar: 400 µm. (TIF) [file pone.0023387.s002.tif]

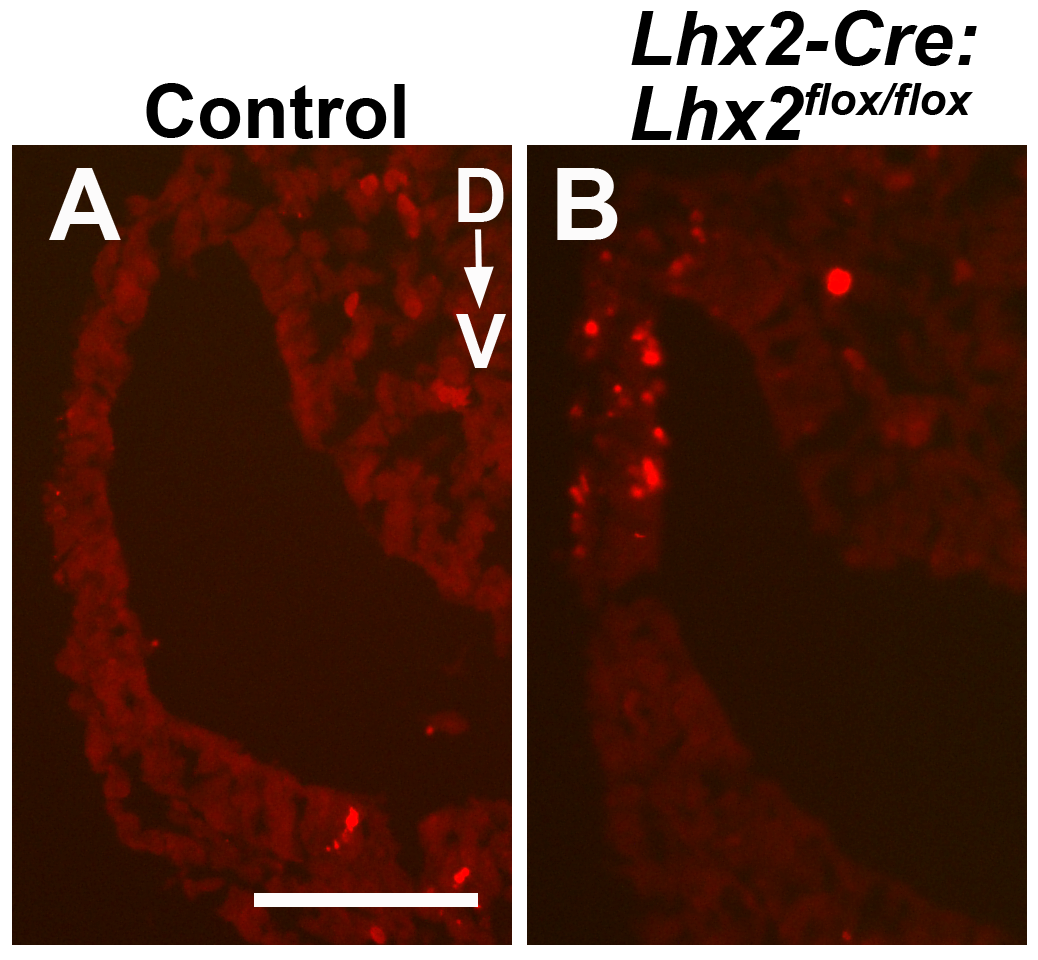

Supplement: Figure S3 — Increased number of apoptotic cells in the mutant optic vesicle. Immunohistochemical analysis of coronal sections of control optic vesicle (A) and mutant (Lhx2-Cre:Lhx2flox/flox) optic vesicle (B) at E9.5 to detect the presence of activated caspase-3. Scale bar: 100 µm. (TIF) [file pone.0023387.s003.tif]

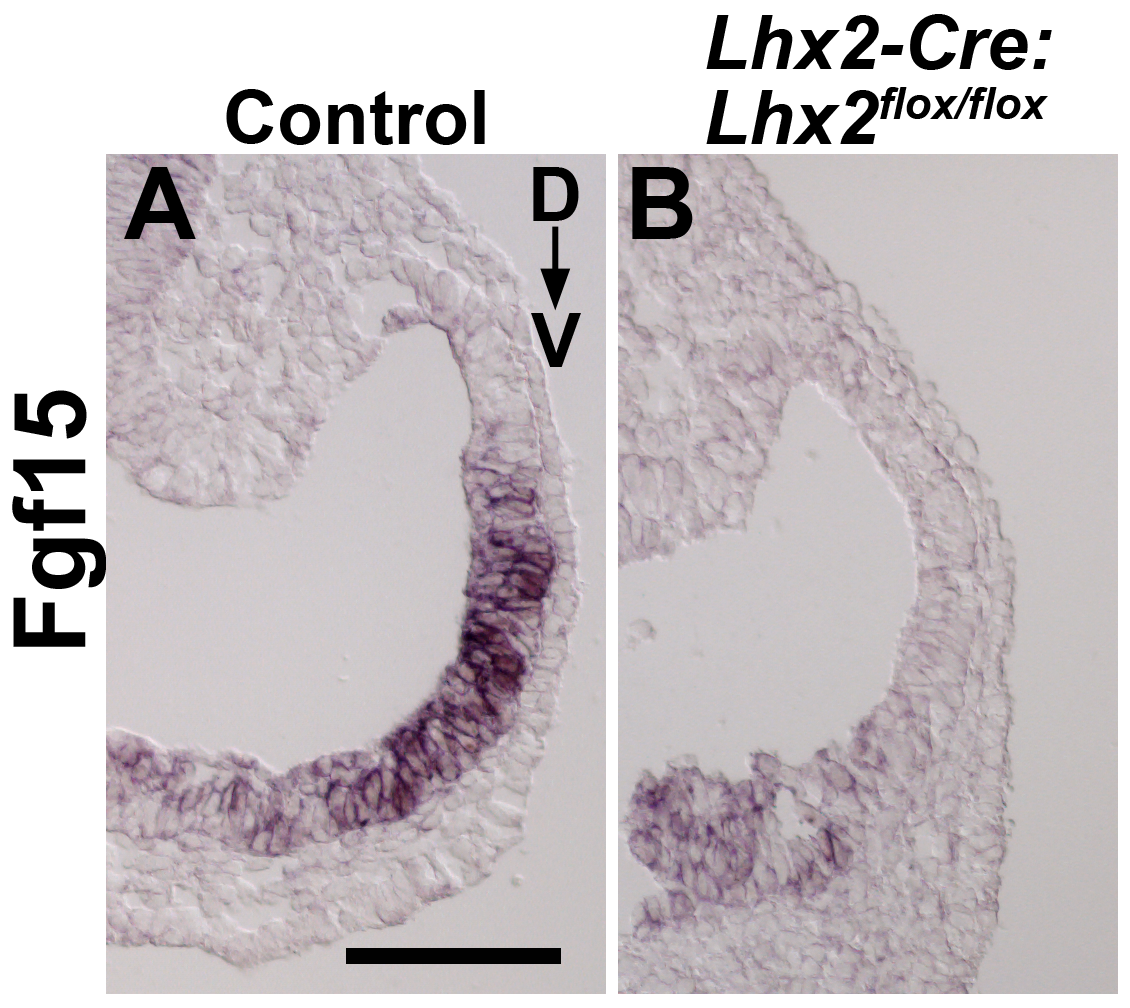

Supplement: Figure S4 — Fgf15 expression is significantly down-regulated in the optic vesicle in the conditional mutant. In situ hybridisation analyses of coronal sections of the optic vesicles in control (A) and mutant (Lhx2-Cre:Lhx2flox/flox) embryos (B) at E9.5 to detect Fgf15 expression. (TIF) [file pone.0023387.s004.tif]

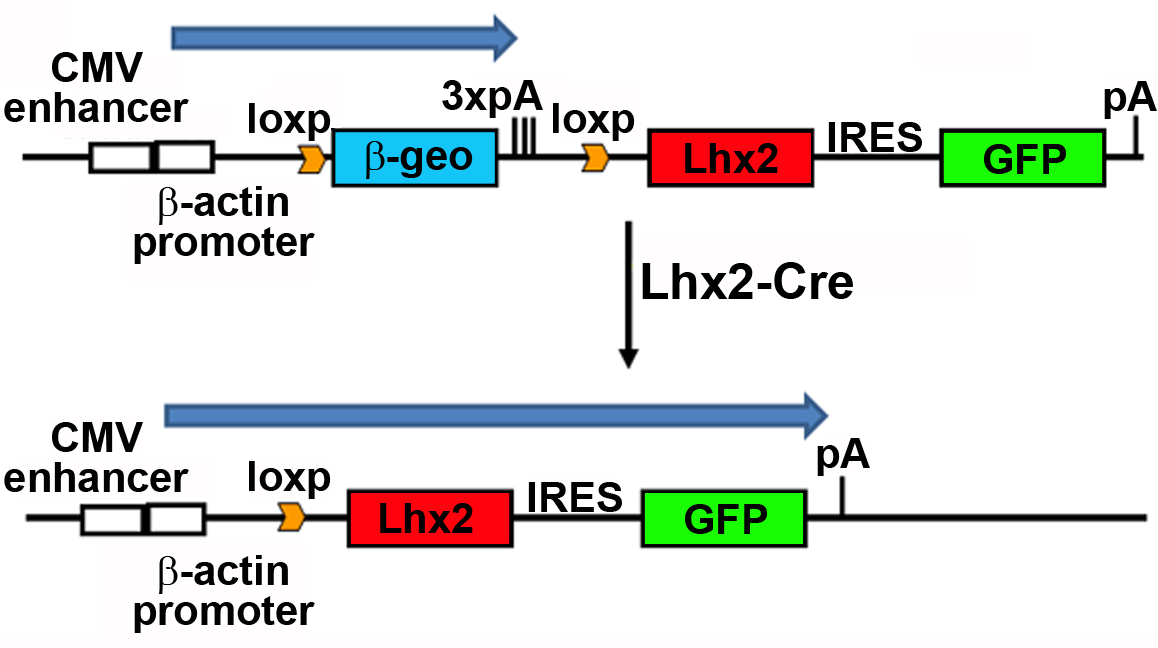

Supplement: Figure S5 — Lhx2 expression is induced following Cre-mediated recombination of the Z/Lhx2-GFP transgene. Schematic representation of the vector used to generate the Z/Lhx2-GFP transgenic mouse strain (upper panel) and the organisation of this vector after Cre-mediated recombination (lower panel). The blue arrows correspond to the mRNA that is generated before and after Cre-mediated recombination of this vector. We utilised an expression system based on the Z/AP double reporter vector developed by Lobe and co-workers [1], where a floxed allele of β-Geo (encoding a β-Gal-Neomycin fusion protein) is followed by an expression cassette consisting of the Lhx2 cDNA, an internal ribosomal entry site (IRES) and green fluorescent protein (GFP) cDNA. Thus, cells expressing Cre recombinase will delete the β-Geo gene and initiate expression of Lhx2 and GFP since the Lhx2-GFP part is placed immediately downstream of the promoter/enhancer. Supplementary reference. 1. Lobe, C., Koop, K., Kreppner, W., Lomeli, H., Gertsenstein, M. and Nagy, A. (1999). Z/AP, a double reporter for cre-mediated recombination. Develop. Biol. 208:281–292. (TIF) [file pone.0023387.s005.tif]
